# Reversal of Tetracycline Resistance by Cepharanthine, Cinchonidine, Ellagic Acid and Propyl Gallate in a Multi-drug Resistant *Escherichia coli*

**DOI:** 10.1007/s13659-020-00280-y

**Published:** 2020-11-03

**Authors:** Darko Jenic, Helen Waller, Helen Collins, Clett Erridge

**Affiliations:** 1 School of Life Sciences, Anglia Ruskin University, East Road, Cambridge, CB1 1PT UK; 2 Diabetes Research Centre, University of Leicester, Leicester General Hospital, Gwendolen Road, Leicester, LE5 4PW UK; 3 Department of Health Sciences, University of Leicester, University Rd, Leicester, LE1 7RH UK

**Keywords:** Antibiotic resistance, Natural products, Phytochemical, Screening, Efflux pump inhibitor

## Abstract

**Abstract:**

Bacterial resistance to antibiotics is an increasing threat to global healthcare systems. We therefore sought compounds with potential to reverse antibiotic resistance in a clinically relevant multi-drug resistant isolate of *Escherichia coli* (NCTC 13400). 200 natural compounds with a history of either safe oral use in man, or as a component of a traditional herb or medicine, were screened. Four compounds; ellagic acid, propyl gallate, cinchonidine and cepharanthine, lowered the minimum inhibitory concentrations (MICs) of tetracycline, chloramphenicol and tobramycin by up to fourfold, and when combined up to eightfold. These compounds had no impact on the MICs of ampicillin, erythromycin or trimethoprim. Mechanistic studies revealed that while cepharanthine potently suppressed efflux of the marker Nile red from bacterial cells, the other hit compounds slowed cellular accumulation of this marker, and/or slowed bacterial growth in the absence of antibiotic. Although cepharanthine showed some toxicity in a cultured HEK-293 mammalian cell-line model, the other hit compounds exhibited no toxicity at concentrations where they are active against *E. coli* NCTC 13400. The results suggest that phytochemicals with capacity to reverse antibiotic resistance may be more common in traditional medicines than previously appreciated, and may offer useful scaffolds for the development of antibiotic-sensitising drugs.

**Graphic Abstract:**

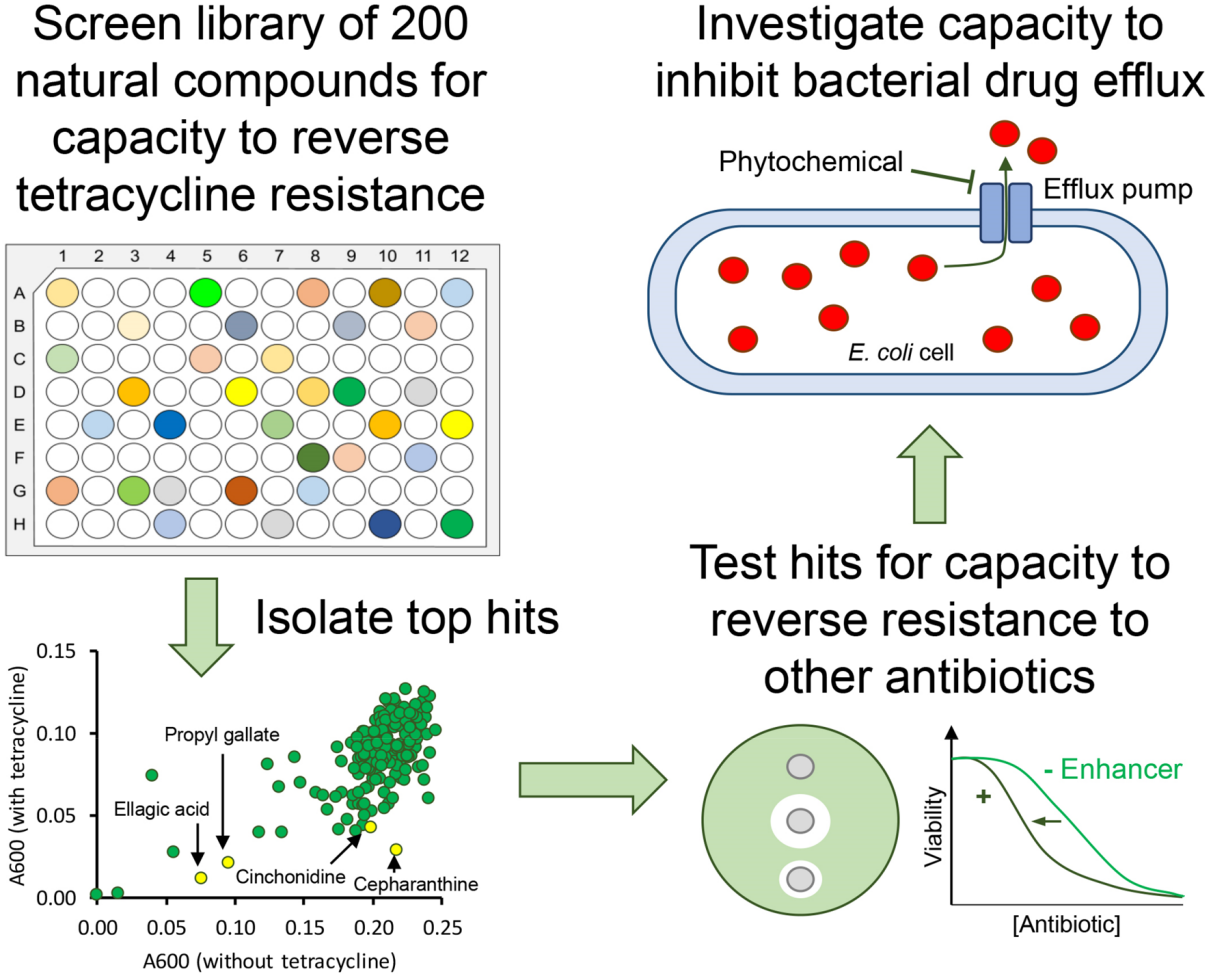

## Introduction

Gram-negative bacteria displaying resistance to multiple classes of antibiotics are increasingly prevalent and difficult to treat in modern healthcare systems [1]. Historically, this issue has been countered by the discovery and development of new classes of antibiotics. However, as only two new classes of antibiotic have been discovered since the 1960s, attention has focussed more recently on the identification of agents which target bacterial mechanisms of resistance [2, 3].

This approach has proven successful for the restoration of utility of some β-lactam antibiotics via lactamase inhibitors, such as clavulanic acid [3]. Some success has also been reported in the identification of compounds with capacity to reverse resistance to various non-lactam antibiotics in Gram-positive bacteria, particularly *Staphylococcus aureus* [4, 5]. However, resistance reversal in Gram negative bacteria has proven significantly more challenging, and comparatively little progress has been made in identifying novel agents with sensitising properties [6].

We therefore sought to identify compounds with capacity to reverse resistance to six antibiotics of different classes using a multi-drug-resistant *Escherichia coli*. Our strategy was based on the hypothesis that since plants have successfully countered the threat of Gram-negative infection for millennia through their production of antibacterial secondary metabolites, it is likely that they have also faced the issue of resistance to these defences via the same resistance mechanisms currently posing a threat to man. This raises the possibility that plant secondary metabolites with potential to combat bacterial resistance mechanisms may have also co-evolved in parallel with compounds with direct bactericidal or bacteriostatic properties, and may therefore have potential as scaffolds for the development of novel classes of therapeutic resistance-reversing drugs.

As small molecules intended for clinical use should also be of low toxicity in mammalian systems, and ideally with potential for rapid translation to human trials, we chose to focus only on compounds isolated from plants with a history of use in man as traditional herbs or medicines. A natural product library comprising 200 such phytochemicals was therefore screened to identify compounds with capacity to reverse resistance using the model multi-drug resistant *E. coli* isolate NCTC 13400. Potential mechanisms for the sensitising properties of the hit compounds, and their impacts on the viability of mammalian cells cultured in vitro, were then explored.

## Results and Discussion

### Primary Screen of 200 Natural Compounds for Reversal of Resistance to Tetracycline

At least four major mechanisms are currently thought to mediate bacterial resistance to antibiotics; namely drug destruction, efflux pumps, target modification and reduced permeability of the cell wall. Although some success has been achieved in the development of drugs with capacity to reverse resistance via the first of these mechanisms (i.e. β-lactamase inhibitors), comparatively little progress has been made in the discovery of molecules with the capacity to block other forms of resistance, and sensitise to other classes of antibiotic, particularly in Gram-negative bacteria [1, 3].

Plants have faced millennia of exposure to Gram-negative pathogens, many of which are likely to have evolved diverse resistance mechanisms to counter bacteriostatic or bactericidal plant compounds [7, 8]. As plants may have also faced selection in turn to develop phytochemical secondary metabolites targeting such mechanisms of resistance, we hypothesised that screening a natural product library may offer potential leads for resistance reversing agents.

**Fig. 1:**
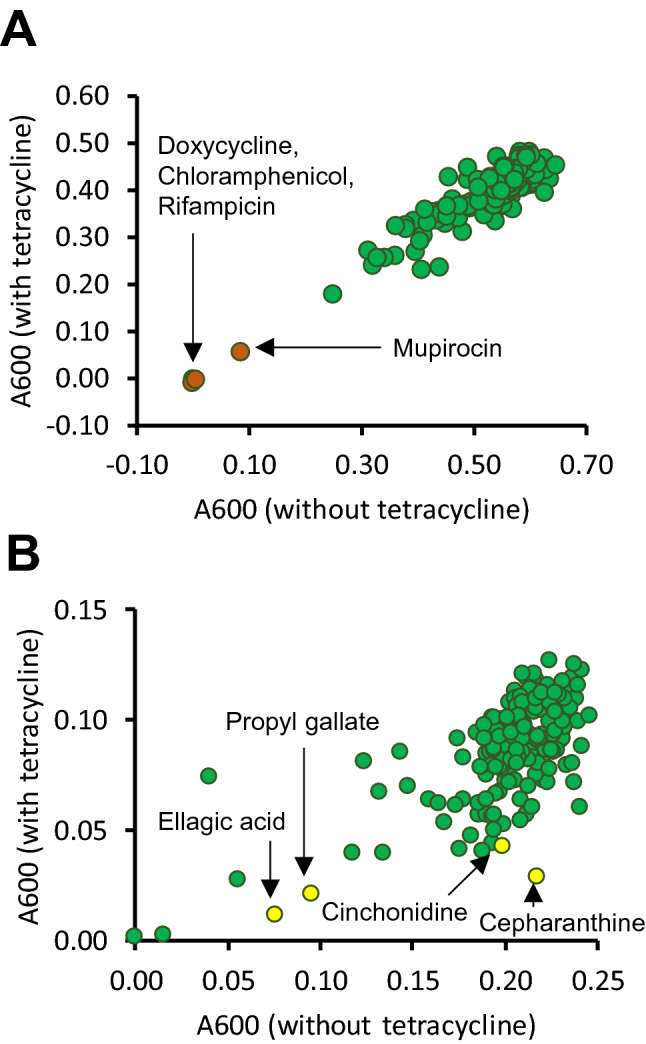
Primary screen of 200 phytochemicals for capacity to reverse tetracycline resistance in *E. coli* NCTC 13400. *E. coli* NCTC 13400 was cultured in microtitre plates in the presence or absence of 0.25× MIC (16 μg/mL) tetracycline with 100 μM of each phytochemical, or vehicle control (1% DMSO). Absorbance at 600 nm was measured at 21 h (**a**) and 5 h (**b**). Each individual point represents one of the tested phytochemicals. Hit compounds chosen for further study are highlighted

The library screened in the present study contains several compounds of natural origin that are used as antibiotics, and three of these (chloramphenicol, doxycycline and rifampicin) fully prevented growth of *E. coli* NCTC 13400 at 100 μM, in the presence or absence of 0.25× minimum inhibitory concentration (MIC) tetracycline (Fig. 1a). None of the non-antibiotic compounds tested achieved the same level of inhibition at the 21 h timepoint. However, a closer inspection of the data from earlier timepoints indicated that several compounds slowed growth to a greater extent in the presence of tetracycline than without, suggesting potential for resistance reversal (Fig. 1b). The four compounds that scored the highest according to this metric (after exclusion of existing antibiotics) were cepharanthine, cinchonidine, ellagic acid and propyl gallate.

**Fig. 2:**
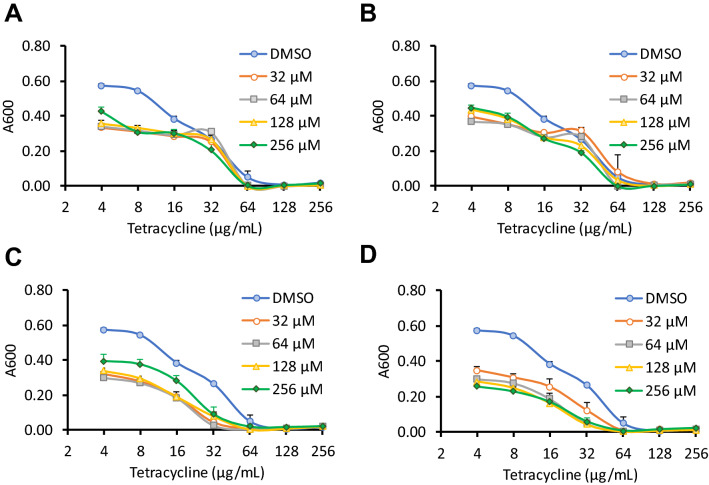
Checkerboard assays of the top four hits from the primary screen. *E. coli* NCTC 13400 was cultured in the presence of doubling dilutions of tetracycline, in combination with doubling dilutions of cepharanthine (**a**), cinchonidine (**b**), ellagic acid (**c**) propyl gallate (**d**), or vehicle control (DMSO). Absorbance was measured at 600 nm at 21 h. Means of three independent experiments ± SD are shown

Checkerboard assays then showed that each of these compounds reduced the MIC of tetracycline by up to twofold at both the 8 and 21 h timepoints (Fig. 2). We hypothesised that the hit compounds may exhibit synergistic or additive effects on resistance reversal, so a mixture of the 4 phytochemicals together (at 64 μM each) was also tested, and this reduced the MIC of tetracycline by fourfold at both timepoints (Table 1).

**Table 1:** MICs and IC_50_ of tetracycline in the presence of maximal inhibitory concentrations of each phytochemical

	MIC (8 h)	MIC (21 h)	IC_50_ (8 h)	IC_50_ (21 h)	P-value*
DMSO	32	64	15.52	25.2	–
Cepharanthine	16	32	6.91	16.0	0.0126
Cinchonidine	16	32	8.80	22.6	0.6840
Ellagic acid	16	32	8.91	15.9	0.0082
Propyl gallate	16	32	6.67	15.4	0.0038
Mix (4 chemical)^a^	8	16	4.05	6.1	< 0.0001

Calculations are based on a phytochemical concentration of 256 μM, except for ellagic acid, which reproducibly exhibited maximal inhibition at 64 μM. MIC and IC_50_ concentrations are given in μg/mL tetracycline

^a^The 4 chemical mix comprises cepharanthine, cinchonidine, ellagic acid and propyl gallate at 64 µM each

*P-values indicate comparisons of IC_50_ values of three independent experiments at 21 h with vehicle alone (2.56% DMSO) by ANOVA with Dunnett’s post test

### Effects of Hit Compounds on Sensitivity of *E*. *coli* NCTC 13400 to Other Antibiotics

**Fig. 3:**
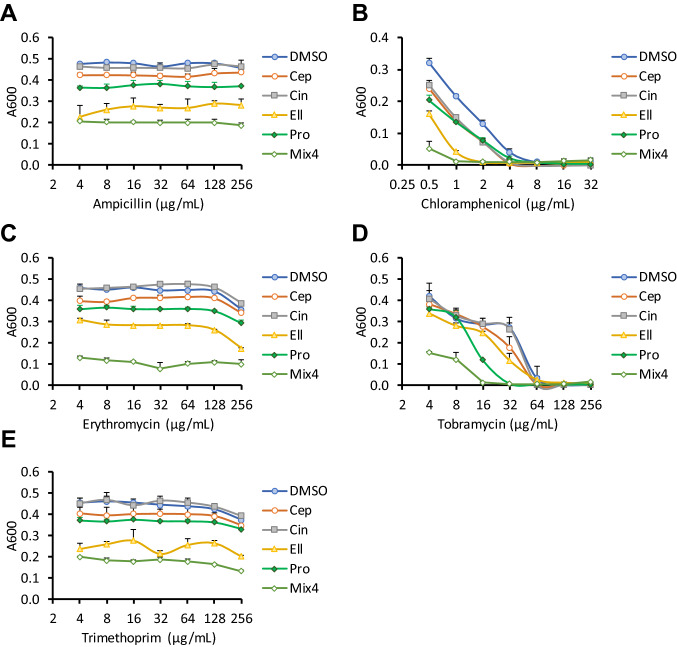
Effects of top hits on sensitivity of *E. coli* NCTC 13400 to other antibiotics. *E. coli* NCTC 13400 was cultured for 21 h in the presence of 64 μM of each phytochemical, vehicle control (0.64% DMSO), and indicated concentrations of ampicillin (**a**), chloramphenicol (**b**), erythromycin (**c**), tobramycin (**d**), or trimethoprim (**e**). Absorbance at 600 nm was measured at 21 h. Means of three independent experiments ± SD are shown. Abbreviations: Ellagic acid (*Ell*), cepharanthine (*Cep*), cinchonidine (*Cin*), propyl gallate (*Pro*), mix of all 4 phytochemicals (Mix4)

The four hit compounds had no impact on the MICs of ampicillin, erythromycin or trimethoprim (Fig. 3). However, the MIC of chloramphenicol was reduced twofold by cepharanthine and cinchonidine, and fourfold by ellagic acid. Propyl gallate also reduced the MIC of tobramycin by twofold, at 21 h (Table 2). A mixture of the 4 phytochemicals together (at 64 μM each) did not impact on the MICs of ampicillin, erythromycin or trimethoprim, but lowered the MIC of tetracycline by fourfold, chloramphenicol by eightfold and tobramycin by fourfold. Disc diffusion assays using each antibiotic or mixture revealed a similar, although less pronounced, pattern of sensitisation (Table 3).

**Table 2:** Effects of hit phytochemicals on sensitivity of *E. coli* NCTC 13400 to antibiotics of other classes

	Ampicillin	Chloramphenicol	Erythromycin	Tobramycin	Trimethoprim
DMSO	> 256	8	> 256	64	> 256
Cepharanthine	> 256	4	> 256	64	> 256
Cinchonidine	> 256	4	> 256	64	> 256
Ellagic acid	> 256	2	> 256	64	> 256
Propyl gallate	> 256	8	> 256	32	> 256
Mix (4 chemical)^a^	> 256	1	> 256	16	> 256
Mix (3 chemical)^b^	> 256	1	> 256	32	> 256

Dose curves of each antibiotic were supplemented with 64 μM of each phytochemical, and MICs were calculated from A600 measured at 21 h. MIC concentrations are given in μg/mL

^a^The 4 chemical mix comprises cepharanthine, cinchonidine, ellagic acid and propyl gallate at 64 µM each

^b^The 3 chemical mix comprises cinchonidine, ellagic acid and propyl gallate at 64 µM each

**Table 3:** Disk diffusion assays of the effects of hit phytochemicals on antibiotic sensitivity in *E. coli* NCTC 13400

	Ampicillin	Chloramphenicol	Erythromycin	Tetracycline	Tobramycin	Trimethoprim
DMSO	NI	20.3 ± 0.6	NI	10.0 ± 0.0	14.0 ± 0.8	NI
Cepharanthine	NI	20.7 ± 1.2	NI	10.0 ± 0.0	14.8 ± 1.5	NI
Cinchonidine	NI	21.7 ± 1.5	NI	10.5 ± 1.0	15.5 ± 1.7	NI
Ellagic acid	NI	24.0 ± 0.0**	NI	11.0 ± 1.2	15.3 ± 2.2	NI
Propyl gallate	NI	23.7 ± 0.6**	NI	13.0 ± 0.8**	18.0 ± 1.8*	NI
Mix (4 chemical)^a^	NI	22.7 ± 1.2*	7.0 ± 1.2	10.3 ± 0.5	15.5 ± 1.3	NI

Zones of clearance are presented as millimetres diameter ± SD (NI = no inhibition). Paper discs were 6 mm in diameter, containing 50 μg antibiotic per disc (except chloramphenicol, which was 1 μg per disc), and 50 nmol of respective individual phytochemicals

^a^The 4 chemical mix comprises cepharanthine, cinchonidine, ellagic acid and propyl gallate at 12.5 nmol each

*P < 0.05, **P < 0.01, ANOVA with Dunnett’s test vs DMSO control. n = 3–4 independent experiments

Two of the top four hits from the present study, or their close derivatives, have been reported previously to exhibit antibiotic potentiating effects. Ellagic acid was reported to potentiate the antibiotics novobiocin, coumermycin, chlorobiocin, rifampicin and fusidic acid in *Acinetobacter baumannii* [9]. Gallic acid, which is structurally similar to propyl gallate, was also shown to potentiate the activities of sulfamethoxazole and tetracycline in *Pseudomonas aeruginosa* [10]. However, to our knowledge, no sensitising properties of cepharanthine or cinchonidine have been reported previously.

### Potential Mechanisms of Action of Hit Phytochemicals

**Fig. 4:**
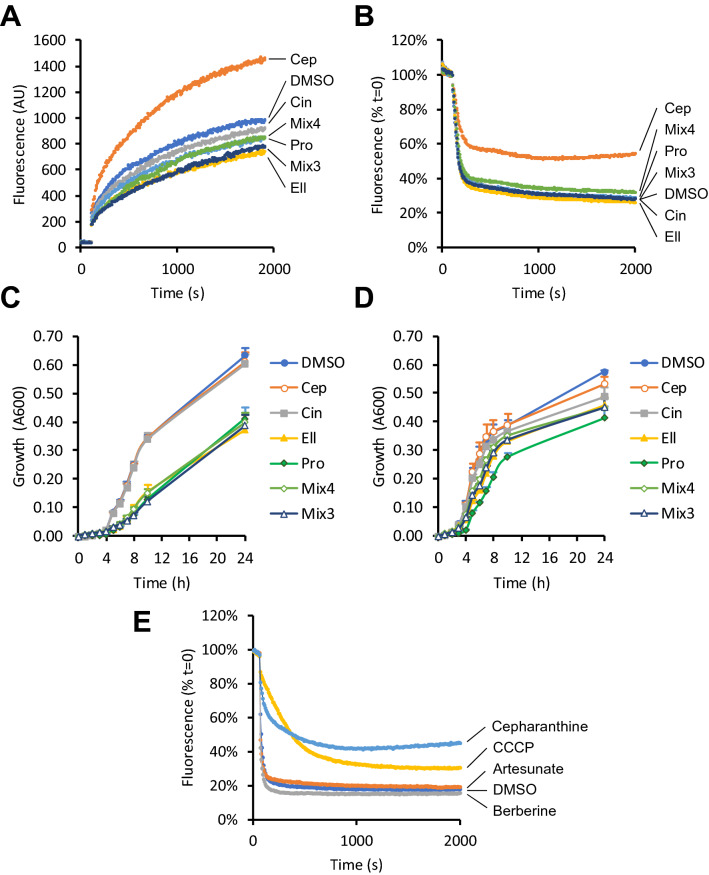
Effects of phytochemicals on uptake and efflux of Nile red in *E. coli* NCTC 13400. For Nile red accumulation assays (**a**), *E. coli* NCTC 13400 was cultured with 64 μM of each phytochemical or vehicle control (0.64% DMSO). After 2 min, 5 μM of the marker Nile red was added. For efflux assays (**b**), CCCP de-energised cells were pre-loaded with Nile red, and re-energised after 2 min with 50 mM glucose. In both cases, fluorescence at 620 nm, which is proportional to cellular content of Nile red, was measured every 5 s for indicated periods. The kinetics of bacterial growth in the absence of antibiotics were also assayed by measuring absorbance at 600 nm for *E. coli* NCTC 13400 (**c**) or *E. coli* DH5-α (**d**), in the presence of 64 μM of each phytochemical, or vehicle control (0.64% DMSO). The effects of cepharanthine, artesunate and berberine (64 µM) on efflux were compared with 50 µM CCCP (a positive control for efflux inhibition) in *E. coli* NCTC 13400 (**e**). Results are representative of at least three independent experiments. Abbreviations: Ellagic acid (*Ell*), cepharanthine (*Cep*), cinchonidine (*Cin*), propyl gallate (*Pro*), mix of all 4 phytochemicals (*Mix4*), mix of Ell, Cin and Pro only (*Mix3*)

Because the hit compounds lowered the MICs of antibiotics from several different classes, we hypothesised that they may act at least partly as inhibitors of multidrug efflux systems. To test this, we first measured accumulation of the lipophilic dye Nile red using *E. coli* NCTC 13400 cells. Cepharanthine markedly accelerated cellular uptake of this dye, consistent with inhibition of efflux, while the other hit compounds and mixtures consistently reduced dye accumulation compared to vehicle control (Fig. 4a). Efflux assays then confirmed that cepharanthine markedly inhibited efflux of Nile red from loaded cells, while the other hit compounds and mixtures had little impact on this pathway (Fig. 4b).

We next tested the hypothesis that the hit compounds may limit cellular metabolism or availability of energy, since these may contribute to resistance mechanisms. In the absence of antibiotics, ellagic acid and propyl gallate both markedly slowed the rate of growth of the test strain, while cepharanthine and cinchonidine had little or no impact on growth rate (Fig. 4c). To test whether the reversal of resistance may be specific to *E. coli* NCTC 13400, the effects of each phytochemical on antibiotic sensitivity were re-examined using the *E. coli* strain DH5-α, which lacks pEK499 and resistance to the tested antibiotics. While ellagic acid generally sensitised this strain to most antibiotics to a similar degree to that observed in *E. coli* NCTC 13400, the other compounds were largely ineffective at reversing resistance to the tested antibiotics in DH5-α (Table 4). In the absence of antibiotics, ellagic acid and propyl gallate had a greater impact on the growth rate of *E. coli* DH5-α than cepharanthine and cinchonidine, but the degree of inhibition was not as pronounced as that seen in *E. coli* NCTC 13400 (Fig. 4d).

**Table 4:** Effects of hit phytochemicals on antibiotic sensitivity of *E. coli* DH5-α

	Ampicillin	Chloramphenicol	Erythromycin	Tetracycline	Tobramycin	Trimethoprim
DMSO	4	4	128	2	16	0.5
Cepharanthine	4	4	128	2	16	1
Cinchonidine	2	4	128	2	16	1
Ellagic acid	2	2	64	1	32	1
Propyl gallate	4	4	128	1	16	2

Dose curves of each antibiotic were supplemented with 64 μM of each phytochemical, and MICs (shown above in μg/mL) were calculated from A600 measured at 21 h

Taken together, these findings suggest that cepharanthine likely mediates its sensitising effects at least partly via its action as an efflux pump inhibitor (EPI), since this compound both accelerated accumulation of Nile red and slowed its efflux from bacterial cells. Notably, although numerous natural EPIs for Gram-positive bacteria have been described [4, 11], very few have been reported for Gram-negative bacteria [2]. Two of the best known examples of such molecules are artesunate, which inhibits AcrAB-TolC in *E. coli* [12], and berberine, which inhibits MexXY-OprM in *P*. *aeruginosa* [13]. Interestingly, both of these compounds are present in the library, but they did not score well for the primary phenotypic endpoint of tetracycline resistance reversal, ranking in 156th, and 65th places, respectively. Further experiments revealed that neither of these compounds inhibits efflux of Nile red from *E. coli* NCTC 13400 (Fig. 4e). Thus, EPI activity against RND type pumps, such as MexXY-OprM and AcrAB-TolC, may not necessarily be a strong indicator of potential to reverse resistance to tetracycline. It is also notable that while artesunate is a proven inhibitor of the AcrAB-TolC system in *E. coli* [12], it did not sensitise to tetracycline in our assays. Taken together, these data suggest that cepharanthine, and the other hits from the present study, are not likely to reverse tetracycline resistance by acting as EPIs for AcrAB-TolC [14].

It is also interesting to note that cepharanthine was reported previously to exhibit EPI activity in cultured mammalian cells, by inhibiting the human P-glycoprotein multi-drug efflux pump [15]. This raises the possibility that the target of cepharanthine activity in *E. coli* may exhibit structural similarity with P-glycoprotein, which is a member of the ATP-binding cassette (ABC)-family of efflux pumps. However, although there are many predicted examples of ABC type pumps in the *E. coli* genome [16], no such pumps of this class are represented on the pEK-499 plasmid [17]. The TetA efflux system, which confers the tetracycline resistance provided by this plasmid, is a member of the structurally distinct major facillitator family of efflux pumps [2]. We note that *E. coli* DH5-α, which lacks the TetA efflux system, was not sensitised to tetracycline by cepharanthine. However, further studies will be required to ascertain more definitively whether the TetA system is a target of cepharanthine EPI activity.

As the other hit compounds did not inhibit Nile red efflux, it is likely that their sensitising properties are conferred via other mechanisms. One possibility is that, as some resistance mechanisms are relatively energy-intensive [18], agents which non-specifically reduce the availability of energy or nutrients could inhibit such mechanisms. Notably, both ellagic acid and propyl gallate reduced the growth rate of *E. coli* NCTC 13400 and *E. coli* DH5-α in the absence of antibiotics. However, further studies will be required to explore this possibility and the mechanism of action of cinchonidine, since this compound had little or no impact on growth in either strain.

### Effects of Hit Compounds on the Viability of Cultured Mammalian Cells

**Fig. 5:**
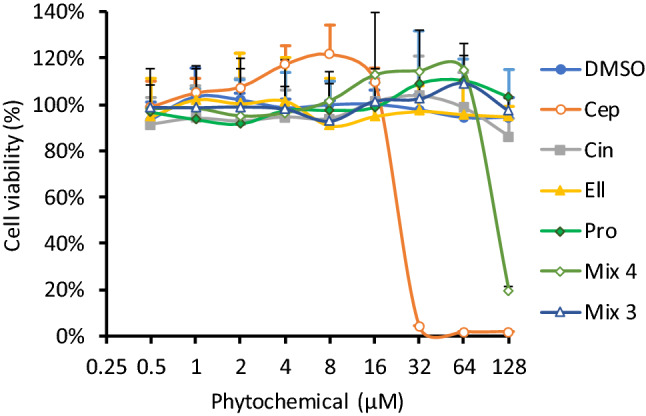
Effects of hit phytochemicals on viability of human HEK-293 cells cultured in vitro. HEK-293 cells were cultured at 2 × 10^4^ cells per well of 96-well plates. After 24 h, cells were treated with indicated concentrations of each phytochemical or DMSO control, and viability was quantified by MTT assay after a further 24 h. Concentrations are given as total phytochemical content for the mixtures. Means of three independent experiments ± SD are shown. Abbreviations: Ellagic acid (*Ell*), cepharanthine (*Cep*), cinchonidine (*Cin*), propyl gallate (*Pro*), mix of all 4 phytochemicals (*Mix4*), mix of Ell, Cin and Pro only (*Mix3*)

To gain preliminary insight into the potential impact of the hit compounds on viability of mammalian cells, MTT assays using the HEK-293 (human embryonic kidney) cell line were performed in vitro. Although there was no significant impact of cinchonidine, ellagic acid or propyl gallate on cell viability up to 128 μM, cepharanthine exhibited toxicity with an IC50 of 24.6 μM (Fig. 5). The mixture of 4 phytochemicals also showed some toxicity at the highest dose tested, presumably due to content of cepharanthine. A mixture of the 3 non-toxic phytochemicals, lacking cepharanthine, was therefore tested, and this mixture showed no effect on viability in this assay at up to 32 μM of each compound. Retesting the 3 compound mixture in the *E. coli* sensitisation assays showed that it had the same effect as the 4 compound mixture on the MICs of the tested antibiotics, except for tobramycin, for which sensitivity was increased by only twofold, rather than fourfold for the 4 compound mixture (Table 2).

Low toxicity in mammalian systems is a key property of any compound intended for use as a scaffold for drug development. It is promising to note that in our HEK-293 system, ellagic acid, cinchonidine and propyl gallate exhibited no toxicity at concentrations up to 128 μM. This is consistent with the established use of ellagic acid and propyl gallate as approved food additives in various territories. Dietary studies in man have also confirmed that doses of up to 500 mg pure ellagic acid are well tolerated with no obvious side effects, and the molecule can reach micromolar concentrations in serum following oral supplementation [19]. Cinchonidine is also likely to be of relatively low toxicity, as it is isolated from the bark of the medicinal plant *Cinchona officinalis*, which has been used to treat fever and malaria for centuries. The low toxicity of these hits supports the hypothesised advantage of screening compounds isolated from traditional medicines, and lends further weight to earlier observations that such compounds are likely to exhibit lower toxicity, and greater ease of translation to trials, than purely synthetic compounds, both of which were key objectives of the present study [20].

Nevertheless, we recognise that cepharanthine exhibited significant toxicity in our HEK-293 system at doses necessary to enhance antibiotic activity. It is possible that this toxicity may relate directly to its activity as an EPI, as numerous attempts have been made to use inhibitors of human multi-drug efflux pumps to reverse chemotherapy resistance of tumours in clinical trials. To date, such trials have typically failed due to excessive toxicity of the EPI used, suggesting that inhibition of certain efflux pumps (such as P-glycoprotein, which is a proven target of cepharanthine [15]), may be inherently toxic to some human cells [21].

In summary, the natural compounds ellagic acid, propyl gallate and cinchonidine are identified as potentially useful sensitisers of *E. coli* carrying the pEK499 plasmid to antibiotics of several classes. Cepharanthine is also identified as a novel inhibitor of efflux in *E. coli*. In terms of potential for further discovery, the relatively fruitful ‘hit rate’ of the primary screen (~ 2%), also suggests that phytochemicals isolated from traditional medicines may be more rich in such compounds than previously appreciated. These molecules could have potential as scaffolds for the development of antibiotic resistance reversing drugs with favourable safety profiles.

## Experimental Section

### General Experimental Procedures

*Escherichia* *coli* NCTC 13400 (Public Health England) was selected for study as it displays resistance to eight different classes of antibiotic, via carriage of the pEK-499 plasmid, which contains ten resistance genes of well-established function [17]. The pEK-499 plasmid is also a common cause of antibiotic resistance in urinary tract infections in the UK [17]. The control strain, lacking resistance to the tested antibiotics, was *E. coli* DH5-α. The phytochemical library used (Puretitre library, Caithness Biotechnologies, UK) comprises 200 compounds of natural origin, and was chosen for screening as it comprises only compounds with a history of either safe oral use in man, or as a component of a traditional herb or medicine.

### Primary Screen for Phytochemicals with Capacity to Reverse Resistance to Tetracycline

Tetracycline was used as the model antibiotic in the primary screen, as the focus of this study was on non-lactamase based resistance mechanisms, and because pEK-499 confers a 32-fold increase in minimum inhibitory concentration (MIC) for tetracycline in *E. coli* strain J53, via expression of the tet(A) gene [17]. An overnight culture of *E. coli* NCTC 13400 was used to inoculate a test culture to a density of ~ 4 × 10^5^ cells/mL in Luria broth (LB) supplemented with 16 μg/mL tetracycline, equivalent to one quarter of the MIC for this antibiotic in this strain. 180 μL of this suspension was then plated in 96-well plates, and 20 μL of each natural compound (at 1 mM with 32 μg/mL tetracycline) was added to each well for a final concentration of 100 μM. 8 wells per plate were used as vehicle controls (1% dimethyl sulphoxide, DMSO). Plates were then incubated at 37 °C, and absorbance at 600 nm was measured at 0, 5, 8 and 21 h using a microplate reader (Tecan). All values were corrected for baseline absorbance by subtraction of the value measured at t = 0 to account for the potential impact of test compounds on absorbance at 600 nm.

Two scores were used to rank the effectiveness of the test compounds in the primary screen. The first score was calculated as the ratio of growth rate between 0 and 5 h with tetracycline to growth rate over the same period without tetracycline. The second score was calculated as the ratio of the growth rate between 5 and 8 h in the presence and absence of tetracycline. The sum of the two scores was then used for the final ranking. Existing antibiotics were removed from the list, and the top four remaining hits were taken forward for replication and further studies.

### Checkerboard Assays and Measurement of Growth Kinetics

The broth microdilution method was used, with doubling dilutions of compound and each antibiotic of interest arranged across a 96-well microplate in a total volume of 150 μL. Plates were incubated at 37 °C, and absorbance at 600 nm was measured at 0, 5, 8 and 21 h using a microplate reader (Tecan). For measurement of growth kinetics in the absence of antibiotics, bacteria were plated with 64 μM of each phytochemical (or 16 μM each for the mixtures), and cultured at 37 °C with measurements of absorbance at 600 nm every hour to 8 h post plating, and also at 10 and 24 h. Baseline (t = 0) absorbance was subtracted from each value to account for pigments associated with the phytochemicals or mixtures. As the hit phytochemicals alone were unable to prevent growth of *E. coli* even at the maximum practicable concentration (1 mM), MICs could not be calculated for these compounds, and therefore fractional inhibitory concentration index (FICI) values could not be determined.

### Bacterial Accumulation and Efflux Assays

Nile red was used as a marker of cellular uptake and efflux in these experiments, as it is a substrate for the *E. coli* AcrAB-TolC efflux system, its polarity is similar to that of many antibiotics and its entry to and exit from the bacterial cell can be conveniently monitored by changes in fluorescence. The method of Bohnert et al. was used, with the following modifications [14]. 1.4 mL of an overnight culture of *E. coli* NCTC 13400 was pelleted (4000*g* for 5 min), then resuspended in phosphate buffered saline (PBS) with 1 mM MgCl_2_ (PBS-M) to an absorbance at 600 nm of 1.0. For accumulation assays, 200 μL of this suspension was plated directly into each well of a 96-well microtitre plate, and phytochemicals were added to a concentration of 64 μM (16 μM of each compound for the mixtures). Fluorescence values for each well were then measured every 5 s for 2 min using excitation of 544 nm and emission of 620 ± 10 nm with a fluorescence microplate reader (Fluostar Omega, BMG Labtech). 20 μL of Nile red stock (1 mM) was then injected into each well to a concentration of 5 μM. Fluorescence values were then measured every 5 s for 40 min. For efflux assays, bacterial suspensions were prepared in PBS-M as above, but were first supplemented with 10 μM carbonyl cyanide m-chlorophenylhydrazone (CCCP, to de-energise cells and halt efflux) for 15 min, before addition of Nile red stock to a concentration of 5 μM. After 3 h incubation at room temperature, cells were pelleted (4000*g* for 5 min), resuspended in 1.4 mL PBS-M, plated at 200 μL per well, and supplemented with phytochemicals as above. Fluorescence values were measured every 5 s for 2 min. Each well was then supplemented with glucose (to 50 mM) to re-energise the cells and restart efflux, and fluorescence measurements were immediately resumed for a further 40 min.

### Mammalian Cell Viability Assays

The HEK-293 cell-line was chosen as a model cell-line to investigate the potential toxicity of the hit compounds in mammalian systems because it is human, not of tumour origin (it is virally transformed), and commonly used to test the effects of new compounds on cell viability [22]. Cells were cultured in Dulbecco’s Modified Eagles Medium (DMEM) with 10% serum and plated at 2 × 10^4^ cells per well of 96-well plates. After 24 h of culture, cells were challenged with phytochemicals at concentrations from 0.5 to 128 μM, or vehicle control (equivalent concentration of DMSO). Cell viability was then measured 24 h later by 3-(4,5-dimethylthiazol-2-yl)-2,5-diphenyltetrazolium bromide (MTT) assay, as described previously [23].

### Statistical Analyses

One-way ANOVA with Dunnett’s post-hoc test was used to compare the means of test conditions with those of the control condition. IC_50_ values, and assessment of potential significance of differences between dose curves, were calculated using GraphPad Prism. Differences were assumed to be significant at P < 0.05.
